# Perspectives About Emergency Department Care Encounters Among Adults With Opioid Use Disorder

**DOI:** 10.1001/jamanetworkopen.2021.44955

**Published:** 2022-01-25

**Authors:** Kathryn Hawk, Ryan McCormack, E. Jennifer Edelman, Edouard Coupet, Nicolle Toledo, Phoebe Gauthier, John Rotrosen, Marek Chawarski, Shara Martel, Patricia Owens, Michael V. Pantalon, Patrick O’Connor, Lauren K. Whiteside, Ethan Cowan, Lynne D. Richardson, Michael S. Lyons, Richard Rothman, Lisa Marsch, David A. Fiellin, Gail D’Onofrio

**Affiliations:** 1 Department of Emergency Medicine, Yale School of Medicine, New Haven, Connecticut; 2 Yale School of Public Health, New Haven, Connecticut; 3 Program in Addiction Medicine, Yale School of Medicine, New Haven, Connecticut; 4 Department of Emergency Medicine, NYU Grossman School of Medicine, New York; 5 Department of Internal Medicine, Yale School of Medicine, New Haven, Connecticut; 6 Geisel School of Medicine at Dartmouth College, Hanover, New Hampshire; 7 Department of Psychiatry, NYU Grossman School of Medicine, New York; 8 Department of Psychiatry, Yale School of Medicine, New Haven, Connecticut; 9 Department of Emergency Medicine, University of Washington School of Medicine, Seattle; 10 Department of Emergency Medicine, Icahn School of Medicine at Mount Sinai, New York, New York; 11 Institute for Health Equity Research, Icahn School of Medicine at Mount Sinai, New York, New York; 12 Department of Emergency Medicine, University of Cincinnati College of Medicine, Cincinnati, Ohio; 13 Center for Addiction Research, University of Cincinnati, Cincinnati, Ohio; 14 Department of Emergency Medicine, Johns Hopkins University School of Medicine, Baltimore, Maryland

## Abstract

**Importance:**

Emergency departments (EDs) are increasingly initiating treatment for patients with untreated opioid use disorder (OUD) and linking them to ongoing addiction care. To our knowledge, patient perspectives related to their ED visit have not been characterized and may influence their access to and interest in OUD treatment.

**Objective:**

To assess the experiences and perspectives regarding ED-initiated health care and OUD treatment among US patients with untreated OUD seen in the ED.

**Design, Setting, and Participants:**

This qualitative study, conducted as part of 2 studies (Project ED Health and ED-CONNECT), included individuals with untreated OUD who were recruited during an ED visit in EDs at 4 urban academic centers, 1 public safety net hospital, and 1 rural critical access hospital in 5 disparate US regions. Focus groups were conducted between June 2018 and January 2019.

**Main Outcomes and Measures:**

Data collection and thematic analysis were grounded in the Promoting Action on Research Implementation in Health Services (PARIHS) implementation science framework with evidence (perspectives on ED care), context (ED), and facilitation (what is needed to promote change) elements.

**Results:**

A total of 31 individuals (mean [SD] age, 43.4 [11.0] years) participated in 6 focus groups. Twenty participants (64.5%) identified as male and most 13 (41.9%) as White; 17 (54.8%) reported being unemployed. Themes related to evidence included patients’ experience of stigma and perceived minimization of their pain and medical problems by ED staff. Themes about context included the ED not being seen as a source of OUD treatment initiation and patient readiness to initiate treatment being multifaceted, time sensitive, and related to internal and external patient factors. Themes related to facilitation of improved care of patients with OUD seen in the ED included a need for on-demand treatment and ED staff training.

**Conclusions and Relevance:**

In this qualitative study, patients with OUD reported feeling stigmatized and minimized when accessing care in the ED and identified several opportunities to improve care. The findings suggest that strategies to address stigma, acknowledge and treat pain, and provide ED staff training should be implemented to improve ED care for patients with OUD and enhance access to life-saving treatment.

## Key Points

**Question:**

What are the experiences and perspectives regarding emergency department (ED) care among US patients with untreated opioid use disorder (OUD) seen in the ED?

**Findings:**

In this qualitative study of 31 individuals with untreated OUD who were seen in the ED and participated in 6 focus groups, participants described stigma, minimization of pain and medical problems, and a need for access to on-demand OUD treatment with referral and ED staff training.

**Meaning:**

The findings suggest that the ability of ED staff to engage patients with OUD may be improved by implementing training on stigma reduction and evidence-based practices to enhance care for these patients.

## Teaser

This qualitative study assesses the experiences and perspectives of individuals with untreated opioid use disorder seen in emergency departments (EDs) in 5 regions of the US.

## Introduction

As US opioid-associated fatalities have continued to increase,^1,2^ emergency departments (EDs) have been recognized as an important venue for initiating treatment for opioid use disorder (OUD) and providing referrals for ongoing care,^3,4,5^ and people seeking treatment for overdose, injection-related infections, withdrawal, and OUD have been increasingly treated in the ED.^6,7,8,9,10,11,12^ Emergency department–initiated buprenorphine with treatment referral is cost-effective and associated with increased 30-day treatment engagement rates compared with standard referral.^13,14^ The treatment gap between people with OUD and those engaged in treatment^15,16^ is associated with stigma, limited patient knowledge about and motivation for treatment, socioeconomic barriers to treatment, and racial and ethnic disparities in access to treatment.^17,18,19,20,21,22,23,24,25,26^ These patient-related factors should be considered when designing interventions to engage patients in the ED. We aimed to explore patients’ perspectives on receiving OUD-related care in the ED to inform future implementation efforts to promote OUD treatment initiation and improve patient-centered care in the ED for those with OUD.

## Methods

### Overview of Project ED Health and ED-CONNECT

This qualitative study was conducted as part of 2 studies. Project ED Health was a hybrid type 3 effectiveness-implementation study designed to investigate differences between an implementation-facilitation strategy and a standard educational dissemination strategy for ED-initiated buprenorphine practice at 4 academic, urban EDs.^27^ ED-CONNECT was a 3-site implementation study investigating the feasibility, acceptability, and effect of introducing an ED-initiated buprenorphine protocol in rural and urban settings with high need, low resources, and different staffing structures.^28^ Project ED Health was approved by Western Institutional Review Board and Project ED CONNECT by Brany Institutional Review Board to identify patients with untreated OUD seen in the ED, enroll for assessment, and follow-up for 30 days. Participants did not receive any specialized treatment intervention. Participants provided verbal informed consent and received a gift card for study participation. This study followed the Standards for Reporting Qualitative Research (SRQR) reporting guideline.^29^

Both studies were grounded in the Promoting Action in Research Implementation in Health Services (PARIHS) framework,^30^ a conceptual framework based on 3 related elements that interact to influence successful implementation of evidence-based practices: evidence (factors related to personal experiences, research findings, and effectiveness), context (readiness of environment for evidence-based practice implementation), and facilitation (strategies to promote targeted change of attitudes, habits, skills, thinking, and working in relation to an evidence-based practice).

### Study Design and Setting

Project ED Health was conducted at 4 EDs, 1 each in Baltimore, Maryland (>70 000 visits per year); New York, New York (>90 000 visits per y); Cincinnati, Ohio (>75 000 visits per year), and Seattle, Washington (>60 000 visits per year). ED CONNECT was conducted at 3 EDs: a critical access hospital in rural New Hampshire (<10 000 visits per year), an urban community ED in Manchester, New Hampshire (35 000 visits per year), and a public hospital in New York, New York (>120 000 visits per year). Patient focus groups were conducted at 4 Project ED Health sites and 2 ED CONNECT sites as part of the formative evaluation.

#### Selection of Participants

Project ED Health focus group participants were recruited among individuals enrolled in Project ED Health during the baseline evaluation period who were willing and able to return to participate in an additional focus group session. ED CONNECT participants were recruited during an ED visit before the formal parent study implementation-facilitation period. Focus group participants were English speaking, were not engaged in OUD treatment when they presented at their ED visit, were not in jail or prison, had capacity to provide informed consent, and either met *Diagnostic and Statistical Manual of Mental Disorders* (Fifth Edition) criteria for OUD (Project ED HEALTH) or endorsed recurrent illicit opioid use and self-identified as having an opioid problem (ED CONNECT).

#### Data Collection and Measurements

Six focus groups, a number typically required to achieve thematic saturation,^31^ were conducted in a nonclinical space between June 2018 and January 2019, with a plan to inform study implementation at each site. Participants anonymously completed a survey capturing demographic characteristics, including age, race and ethnicity (to assist in the evaluation of the applicability of study findings to the EDs), education, marital status, and employment history. Focus groups were facilitated by 3 of us (K.H., R.M., and G.D.), who identified themselves to the groups as emergency physician researchers with training in addiction medicine and who were not responsible for clinical care of the participants. Focus groups were semistructured and 1 hour in length. Facilitators used an interview guide with specific prompts related to the PARIHS elements of evidence, context, and facilitation (Box).^27,30^ Interviews were audio recorded and transcribed. Within quotations, trade names of drugs were replaced with generic names in the reporting of results.

Box. **Semistructured Interview Guide Grounded in the Promoting Action on Research Implementation in Health Services Framework^a^**

1. To get started, can you tell me generally about your understanding of how much there is a need for treatment of opioid use disorder?
  Prompt:
    To what extent do you think this addiction impacts yourself or others who come to this ED?
2. Can you tell me, what is your understanding of the science regarding the relevance of use of buprenorphine for the treatment of opioid use disorder in general? What about for starting buprenorphine in the ED?
  Prompts:
    What do you think makes this relevant for you or others seen in the ED?
    What do you think makes this less relevant for you or others seen in the ED?
3. What about your doctors or other health care providers? What do you think they know about this? How much do they talk to you about your opioid use and treatment options?
  Prompts:
    What kinds of conversations do you have about these issues?
    What about buprenorphine specifically?
    What about referral for medication for your addiction?
4. What if you were to design a system for starting buprenorphine for individuals with an opioid use disorder when they come to the ED? What would it look like?
  Prompts:
    When would you start the buprenorphine?
    How open would you be to taking a medication?
    What else would be helpful?
    What kind of information would you need?
    What about continuing medication through a program? Where would you go?

Abbreviation: ED, emergency department.

^a^
Trade names of drugs were replaced with generic names.

#### Data Analysis

Survey response data were summarized using descriptive statistics generated using SPSS, version 28 (IBM Corporation). A rapid analysis process^32^ was used with iterative analysis and triangulation to develop a preliminary understanding of patients’ perspectives to inform clinical practice change in near real time. An inductive coding process was used to identify ideas and themes within and across focus groups that overlapped with conduction of focus groups. At least 3 members of the analysis team (K.H., E.C., N.T., and P.G.) of racially and ethnically diverse, multidisciplinary researchers with experience in qualitative and ED-based addiction research independently reviewed each transcript to develop and refine the codebook. Coding of each transcript was discussed line by line; consensus and thematic saturation were reached. New codes and themes emerged organically from the text in the tradition of grounded theory.^33^ An audit trail was maintained. Atlas.ti software, version 8.0 (Atlas.ti Scientific Software Development)^34^ was used to facilitate data organization and retrieval. With use of constant comparison methods and thematic analysis, common patterns were identified within the data and were organized into themes.^35^ Themes were then generated based on coded quotations through discussion using the PARIHS framework and its interrelated elements,^30^ and findings that emerged from the data were mapped into the PARIHS framework core elements.^36^

## Results

### Participant Characteristics

A total of 31 individuals participated in 6 focus groups (range, 2-9 per focus group). Of these individuals, 20 (64.5%) identified as male, 0 as Asian, 11 as Black (35.5%), 2 (6.5%) as Hispanic, 5 (16.1%) as Native American/American Indian, 29 (93.5%) as Non-Hispanic, 13 (41.9%) as White, and 2 (6.5%) as other; 4 (12.9%) did not provide their race (Table 1).

**Table 1.** Focus Group Participant Characteristics by Emergency Department Site of Enrollment^a^

Characteristic	Participants, No. (%)						
	Site 1	Site 2	Site 3	Site 4	Site 5	Site 6	Total
Participants	5 (16.1)	9 (29.0)	6 (19.4)	4 (12.9)	5 (16.1)	2 (6.5)	31 (100)
Sex							
Female	0	3 (9.7)	2 (6.5)	3 (9.7)	1 (3.2)	2 (6.5)	11 (35.5)
Male	5 (16.1)	6 (19.4)	4 (12.9)	1 (3.2)	4 (12.9)	0	20 (64.5)
Age, mean (SD), y	48.5 (9.4)	35.6 (12.5)	41.4 (14.2)	43.7 (9.7)	40.9 (4.9)	50.2 (5.7)	43.4 (11.0)
Race^b^							
Asian	0	0	0	0	0	0	0
Black	2 (6.5)	4 (12.9)	1 (3.2)	1 (3.2)	3 (9.7)	0	11 (35.4)
Native American/American Indian	0	1 (3.2)	1 (3.2)	2 (6.5)	1 (3.2)	0	5 (16.1)
White	2 (6.5)	5 (16.1)	0	3 (9.7)	1 (3.2)	2 (6.5)	13 (41.9)
Other	1 (3.2)	0	0	1 (3.2)	0	0	2 (6.5)
Not provided	0	0	4 (12.9)	0	0	0	4 (12.9)
Ethnicity							
Hispanic	0	0	1 (3.2)	0	1 (3.2)	0	2 (6.5)
Non-Hispanic	5 (16.1)	9 (29.0)	5 (16.1)	4 (12.9)	4 (12.9)	2 (6.5)	29 (93.5)
Education							
Less than high school	1 (3.2)	0	2 (6.5)	2 (6.5)	3 (9.7)	0	8 (25.8)
High school or GED	0	6 (19.4)	0	1 (3.2)	2 (6.5)	0	9 (29.0)
Some college	2 (6.5)	3 (9.7)	2 (6.5)	1 (3.2)	0	2 (6.5)	10 (32.3)
College graduate or higher	2 (6.5)	0	0	0	0	0	2 (6.5)
Not provided	0	0	2 (6.5)	0	0 (3.2)	0	2 (6.5)
Employment							
Employed	0	2 (6.5)	2 (6.5)	0	1 (3.2)	1 (3.2)	6 (19.4)
Temporary leave	1 (3.2)	0	0	0	0	0	1 (3.2)
Unemployed, looking	1 (3.2)	6 (19.4)	2 (6.5)	3 (9.7)	4 (12.9)	1 (3.2)	17 (54.8)
Retired	1 (3.2)	0	0	0	0	0	1 (3.2)
Disability	2 (6.5)	1 (3.2)	2 (6.5)	1 (3.2)	0	0	6 (19.4)
Marital status							
Married	0	0	2 (6.5)	2 (6.5)	0	0	4 (12.9)
Living with partner	0	3 (9.7)	1 (3.2)	0	1 (3.2)	0	5 (16.1)
Widowed	1 (3.2)	0	1 (3.2)	0	0	0	2 (6.5)
Divorced	1 (3.2)	0	1 (3.2)	0	1 (3.2)	1 (3.2)	4 (12.9)
Separated	0	0	0	0	0	0	0
Never married	3 (9.7)	5 (16.1)	1 (3.2)	2 (6.5)	3 (9.7)	1 (3.2)	15 (48.4)
Not provided	0	1 (3.2)	0	0	0	0	1 (3.2)

Abbreviation: GED, general educational development.

^a^ Sites 1 through 4 were academic centers; site 5, a public safety net hospital; and site 6, a rural critical access hospital.

^b^ Multiple participants selected more than 1 race, with 2 selecting other.

### Themes

Six main themes emerged across the 3 domains of the PARIHS framework (Figure and Table 2). Themes in the evidence element included the experience of stigma and minimization of pain and medical problems. Context-related themes were that the ED was not seen as a source of OUD treatment and that readiness to initiate treatment was multifaceted, time sensitive, and related to internal and external patient factors. Facilitation themes included the need for on-demand treatment and for ED staff training.

**Figure.**
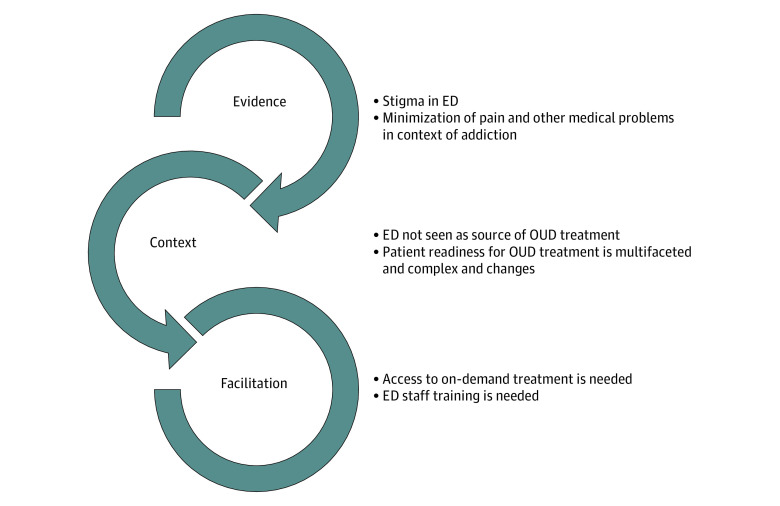
Model Based on the Promoting Action in Research Implementation in Health Services Framework

**Table 2.** Illustrative Quotes Organized by PARIHS Element

PARIHS domain, theme	Illustrative quote (focus group)^a^
Evidence	
Stigma	“Once the hospital finds out that is something that’s part of your lifestyle, 9 times out of 10, you’re treated completely differently. Growing up, we’re always taught that we’re supposed to be open with our medical provider so that they can give us the best care possible, and just a lot of places treat you like dirt.” (4) “Why is she treating me like that? Was it because I had a drug problem?…I don’t care who it is that’s in there, they are at their worst.” (1)
Minimization of pain and medical problems	“You go to like the ER. I got a surgery for a hernia. You know, it’s down by my groin area and painful. They’re like, ‘Oh, well, we’ll write you a prescription of ibuprofen.’ I’m like, ‘Dude, this is serious pain.’” (5) “I was extremely defensive because I’ve been—basically, I’ve been taught to be that way by other emergency room doctors….I have had emergency room doctors tell me that I should be ashamed of myself…and just shamed, shamed and belittled and made to feel, you know, as though my pain is not—is not real.” (6)
Context	
ED not seen as source of substance use disorder treatment	“The staff here, really wasn’t conductive for seeking treatment. It doesn’t seem like a high priority on their list.” (1) “I asked multiple times for help and, they—sometimes would just give me more drugs. They’d give me a shot of hydromorphone and send me out.” (6)
Patient readiness to access OUD treatment is multifaceted and variable	“If I don’t have a bed today, the odds are—I’m not going to go because I’m going to have to keep using something else today.” (3) “I’m telling them that I’m sick, and they giving me nothing—you have to wait til you get upstairs—I would’ve bounced, 2.5 seconds, but I was adamant about it, and I just stuck it out.” (5)
Facilitation	
On-demand treatment	“I came back the second night because they said, ‘Come back if you still have withdrawal symptoms’ because they didn’t give me any buprenorphine [on my first visit]. So, I came back the second night…and he gave me the buprenorphine and it was like an instant cure. I’m like, ‘Oh my God! Thank you!’” (6) “I see it as a starting point, like on a board game or something. If you don’t know where to go, where you could go find help for this, the first thing you think of is the hospital, so people go to the emergency room seeking help.” (2)
Need for training	“Until we can get people to walk in and say, ‘You’re worth living for,’ there’s no real window for us to even look out of.” (3) “You’re tricking your brain into thinking that you doing drugs is something that’s going to prolong the human race….Your brain is not going to want you to stop that. You can’t just stop doing drugs. You can’t. You can’t stop doing drugs, and they don’t understand that.” (2)

Abbreviations: ED, emergency department; ER, emergency department; OUD, opioid use disorder; PARIHS, Promoting Action on Research Implementation in Health Services.

^a^ Trade names of drugs were replaced with generic names.

#### Stigma

When participants were asked about previous experiences in the ED in relationship to their history of opioid use, many provided histories of stigmatizing, often traumatic, experiences that shaped their overall perception of ED care. An individual in focus group 6 stated, “I’m being shamed and treated horribly. And then when the doctor treats you like that, then the nurses aren’t nice to you. They’re all like, oh God, here she comes again.”

Many participants noted that those prior experiences shaped an individual’s willingness to seek care in the ED for general medical problems and addiction. The ED was often described as a last resort for care and a place in which patients’ needs were often unmet. An individual in focus group 2 stated, “It’s one of your last options because you have no options already, so you come into this place. I know when I’m coming here, it’s really probably not going to work because they look at this whole epidemic, all of us, as people who are just almost a waste of their time.”

Of importance, several participants reported recent positive interactions with ED staff and fewer experiences with stigma when seeking care during recent ED visits, highlighting the importance of the ED staff approach in patient experience. An individual in focus group 4 stated, “Just the fact that the people that I spoke with when I was here actually were happy when they were talking to me, they were willing to listen to what I had to say and didn’t have any judgement towards what I said, it made me feel safe being here.”

#### Minimization of Pain and Medical Problems

Many participants described a perception that ED staff believed that they were there just to get pain medication or were “drug-seeking,” noting that their pain was not taken seriously and was rarely adequately addressed. Participants reported a perception that their pain and other medical complaints were minimized if there was any acknowledgment of drug use. An individual in focus group 2 stated, “I broke my nose a month and a half ago, and I fractured my orbital bone. They sent me home with acetaminophen 800 [mg] because I had heroin in my system…they don’t take it seriously.”

Participants also described being frustrated at feeling penalized or like they were perceived as less deserving of care because of their opioid use. An individual in focus group 5 stated, “Why did he have to go home in pain because of this nurse, like it’s totally unrelated. And she was like ‘Well, I don’t know if I’ll give you the cream because you’re sedated.’ ”

Several participants specifically highlighted a desire for more than just pain medication when seeking medical care for undiagnosed or untreated pain. An individual in focus group 3 stated, “I don’t need you all for narcotics. This ain’t where I’d go to get narcotics. This is where I go to get help the legal, the right, the way it’s supposed to be, the American way, but doctors and nurses and—when you walk in, and the first thing they do is look at you like you’re a dope fiend.”

#### ED Not Seen as a Source of OUD Treatment

Participants noted that, in general, the ED did not seem equipped with the knowledge, training, or resources to effectively address the treatment needs of patients with OUD. One participant in focus group 4 described a prior ED visit in which her mother accompanied her and begged the ED doctor for anything that would help treat her heroin withdrawal symptoms: “The nurse and doctor looked at her like she was crazy, and they’re like, ‘Nah, she just has to deal with it.’ ”

Participants also noted challenges with identifying outpatient treatment resources and identified a need for more than the “sheet of paper,” which often contained outdated or unhelpful information, programs that did not match the patients’ needs, or were inaccessible to patients based on insurance status or waitlists. An individual in focus group 1 stated, “They give you this sheet of paper, say, ‘Hey, it’s on you now…’ It gives me the feeling that they don’t care about me.”

Other participants specifically contrasted recent ED experiences with their expectations and prior ED experiences in which needs were not met. An individual in focus group 3 stated, “Y’all saved my life. It’s just real hard for me to talk about sometime because I’m just grateful. I can’t believe that I’m sitting here telling my story. Excuse me. It’s scary. It was devastating. I got here, and I met a whole bunch of—boy, so many people—I couldn’t believe it—that was genuinely concerned about my health instead of me being a dope fiend.”

#### Patient Readiness to Access OUD Treatment Is Multifaceted and Time Sensitive

Participants described that the decision to initiate treatment was complex, with multiple factors impacting readiness to initiate or engage with OUD treatment. They acknowledged that readiness to initiate treatment was dependent on a combination of internal readiness and external factors such as immediate access to addiction care and was sometimes but not always prompted by a significant event such as an overdose or injury. An individual in focus group 3 stated, “If that doctor said, ‘Look, John [name changed], we have a detox up on the third floor, are you willing to go?’ I would’ve said, ‘Sign me up right now.’...The first couple times I overdosed, I don’t know if I was truly ready for help. It scared the shit out of me when you die, but I really wasn’t ready. I’m just being honest. I wasn’t ready.”

Even when they were not interested in engaging in treatment, several participants expressed gratitude toward ED staff who were willing to discuss treatment options and provide referrals as needed. An individual in focus group 4 stated, “It wasn’t something that felt like [it] was pressured on to you, which I believe, in recovery, can be kind of detrimental in people’s recovery because it’s kind of like you can’t make a horse drink water.”

#### On-demand Treatment

Participants noted challenges with accessing treatment, including delays. Some noted a lack of familiarity with treatment options and how to access them, and others offered that ED staff discussing treatment initiation and linkage in the ED would help enhance confidence around engaging in treatment. An individual in focus group 6 stated, “I need treatment, and they just give you a list and send you out and then you call the list and, you know, you don’t have insurance. I needed more guidance.”

Several patients identified the inability to tolerate opioid withdrawal as a large barrier to accessing treatment, particularly when there were delays accessing an outpatient appointment or intake evaluation. An individual in focus group 5 stated, “But buprenorphine, I think it would be good to start it in the ER [emergency room] because at least you know when you got out you could also get this. Next day you follow up.”

#### ED Staff Training Is Needed

Deficiencies in ED staff knowledge and treatment of OUD as a medical disease was identified by many patients. Participants described hearing from ED staff that addiction is a choice, not a disease, and that patients can simply choose to stop using opioids without effective treatment. An individual in focus group 2 stated, “There are some doctors that still don’t believe that it’s a disease. They believe it’s a choice.”

Participants also identified gaps in knowledge about effective management of OUD and described interactions in which buprenorphine was discussed but not initiated or provided by the ED clinician because they didn’t have the right training needed to prescribe buprenorphine. An individual in focus group 3 stated, “[I said,] ‘Is there anything you can do? Can you give me some diazepam? Can you give me buprenorphine? Can you give me anything?’ He said, ‘I can’t give you diazepam because that’s habit forming. I can’t give you buprenorphine because I’m not licensed to do it.’”

## Discussion

This qualitative study evaluated the perspectives and experiences of patients with OUD regarding receiving care in the ED. Key themes that emerged focused on the experience of stigma, minimization of medical needs by ED staff, and the ED not being seen as a source of OUD treatment. Participants indicated a desire to be treated with respect and wanted their pain and their autonomy to be acknowledged. Some comments revealed specific opportunities to facilitate improved care for patients with OUD seen in the ED, including offering on-demand treatment and ED staff training on stigma, OUD, and OUD treatment. Given recent advances in resources and support for treating OUD in EDs^4,37,38,39^ they should be capable of making these changes; however, implementation will require optimized training to reduce stigma and enhance care for patients with OUD.

The study findings are consistent with research highlighting the experience of people who use drugs.^18,40,41,42,43^ In one study, people who inject drugs reported feeling stigmatized by first responders and hospital staff and associated stigmatization with delayed and substandard medical care.^41^ A recent qualitive study^25^ of patients receiving care in the ED after opioid overdose identified a number of factors related to consideration of treatment, including provider communication skills, stigma, availability of ED resources, and support for unmet basic needs. Reports of stigma by patients with OUD in the ED are consistent with studies of attitudes of ED clinicians, in which some clinicians identified patients with OUD as a more challenging and less satisfying patient population to treat.^23,42^

Although many participants reported prior stigmatizing experiences in the ED, a few also noted positive experiences in which ED staff expressed understanding of OUD, with willingness to discuss and provide treatment. Although this experience may be associated with participants being recruited from sites participating in an implementation study of ED-initiated buprenorphine, it suggests a shift toward patient-centered OUD care in the ED. Implementation studies examining ED-based OUD treatment programs have highlighted the use of such patients’ stories as a key strategy to engage ED clinicians and provide feedback on positive patient outcomes.^22,44^ Gaps in the training of ED clinicians on the treatment of OUD have been acknowledged.^22,24,39^ The American College of Emergency Physicians has supported the development of online trainings and webinars, including ED-specific training on the Drug Addiction Treatment Act of 2000,^45^ the Emergency Quality Network Opioid Initiative online learning collaborative, and a consensus guideline on the treatment of OUD in the ED.^38,46,47^

A desire for individual autonomy and respect from health care professionals seemed to drive the needs and preferences of many participants. Although some described experiences of accessing treatment and the importance that it be available when needed, others also described ambivalence about accessing treatment at different time points. This finding highlights the need for ED-based interventions to be patient-centered and to incorporate strategies to explore and enhance patient understanding of potential motivation for behavior change. One example is the Brief Negotiation Interview, a short patient-centered discussion that incorporates feedback and advice to enhance patient motivation and assist the patient in making a positive change regarding substance use.^48,49^ Enhancing ED staff’s ability to participate in these patient-centered discussions may facilitate patient readiness to engage with treatment or use harm reduction strategies while enhancing the understanding of ED staff about OUD and the importance of immediate access to life-saving medication.^25^ In addition, through building relationships with outpatient clinicians and leveraging support staff, the ED can play a critical role in supporting patients who need assistance navigating the treatment system.

### Limitations

This study has limitations. This qualitative research may not be generalizable to other groups of patients with OUD, particularly in the era of COVID-19. Second, focus groups were scheduled to co-occur with study investigator site visits, which may have introduced selection bias. The study findings may have been subject to social desirability bias, although we sought to minimize this through the exclusion of study and clinical staff from focus groups and use of outside focus group facilitators. Our themes were determined in part by the interview guide and the overall objective of the parent studies. One of us (K.H.) facilitated focus groups and was part of the coding team. Potential effects of this dual role were mitigated by having 3 coding team members who did not conduct any focus groups.

## Conclusions

In this qualitative study, patients with OUD frequently reported feeling stigmatized and minimized when accessing care in the ED and identified several opportunities to improve care. The findings suggest that strategies to address stigma, acknowledge pain and patient autonomy, and enhance ED staff knowledge about addiction and OUD treatment should be implemented and evaluated. Future studies may assess whether applying knowledge gained from examining the perspectives and experiences of patients with untreated OUD seen in the ED is associated with reducing opioid-associated fatalities in the US.
